# Successful nonoperative management by endoscopic and percutaneous drainage for penetrating pancreatic duct injury: a case report

**DOI:** 10.1186/s13256-020-02647-8

**Published:** 2021-02-03

**Authors:** Hiroki Kanno, Yusuke Hirakawa, Masafumi Yasunaga, Ryuta Midorikawa, Shinichi Taniwaki, Yoshihiro Uchino, Shin Sasaki, Satoki Kojima, Yoriko Nomura, Goichi Nakayama, Yuichi Goto, Toshihiro Sato, Ryuichi Kawahara, Hisamune Sakai, Hiroto Ishikawa, Toru Hisaka, Koji Okuda

**Affiliations:** Department of Surgery, Kurume University School of Medicine, 67 Asahi-machi, Kurume, Japan

**Keywords:** Penetrating pancreatic trauma, Duct injury, Nonoperative management

## Abstract

**Background:**

Pancreatic trauma is a rare condition with a wide presentation, ranging from hematoma or laceration without main pancreatic duct involvement, to massive destruction of the pancreatic head. The optimal diagnosis of pancreatic trauma and its management approaches are still under debate. The East Association of Surgery for Trauma (EAST) guidelines recommend operative management for high-grade pancreatic trauma; however, several reports have reported successful outcomes with nonoperative management (NOM) for grade III/IV pancreatic injuries. Herein, we report a case of grade IV pancreatic injury that was nonoperatively managed through endoscopic and percutaneous drainage.

**Case presentation:**

A 47-year-old Japanese man was stabbed in the back with a knife; upon blood examination, both serum amylase and lipase levels were within normal limits. Contrast-enhanced computed tomography (CT) showed extravasation of the contrast medium around the pancreatic head and a hematoma behind the pancreas. Abdominal arterial angiography revealed a pseudo aneurysm in the inferior pancreatoduodenal artery, as well as extravasation of the contrast medium in that artery; coil embolization was thus performed. On day 12, CT revealed a wedge-shaped, low-density area in the pancreatic head, as well as consecutive pseudocysts behind the pancreas; thereafter, percutaneous drainage was performed via the stab wound. On day 22, contrast radiography through the percutaneous drain revealed the proximal and distal parts of the main pancreatic duct. The injury was thus diagnosed as a grade IV pancreatic injury based on the American Association for the Surgery of Trauma guidelines. On day 26, an endoscopic nasopancreatic drainage tube was inserted across the disruption; on day 38, contrast-enhanced CT showed a marked reduction in the fluid collection. Finally, on day 61, the patient was discharged.

**Conclusions:**

Although the EAST guidelines recommend operative treatment for high-grade pancreatic trauma, NOM with appropriate drainage by endoscopic and/or percutaneous approaches may be a promising treatment for grade III or IV trauma.

## Background

Pancreatic trauma is a rare condition for which the optimal diagnosis and management are still under debate. The American Association for the Surgery of Trauma (AAST) scale is the most common grading system for pancreatic injuries [1], classifying them into grades I to V depending on the extent of injury, main pancreatic duct involvement, and anatomical location. Grade I/II is defined as hematoma or laceration without main pancreatic duct involvement; grade III is defined as pancreatic body or tail injury with main duct involvement; and grade IV is defined as pancreatic head injury with main duct involvement. Grade V is defined as massive destruction to the pancreatic head. The East Association of Surgery for Trauma (EAST) guidelines recommend operative management for grade III and IV pancreatic trauma [2]; however, several studies have demonstrated that grade III/IV pancreatic injuries can be successfully treated with nonoperative management (NOM) [3–5]. Herein, we report a case of grade IV pancreatic injury that was nonoperatively managed through endoscopic and percutaneous drainage.

## Case presentation

**Fig. 1:**
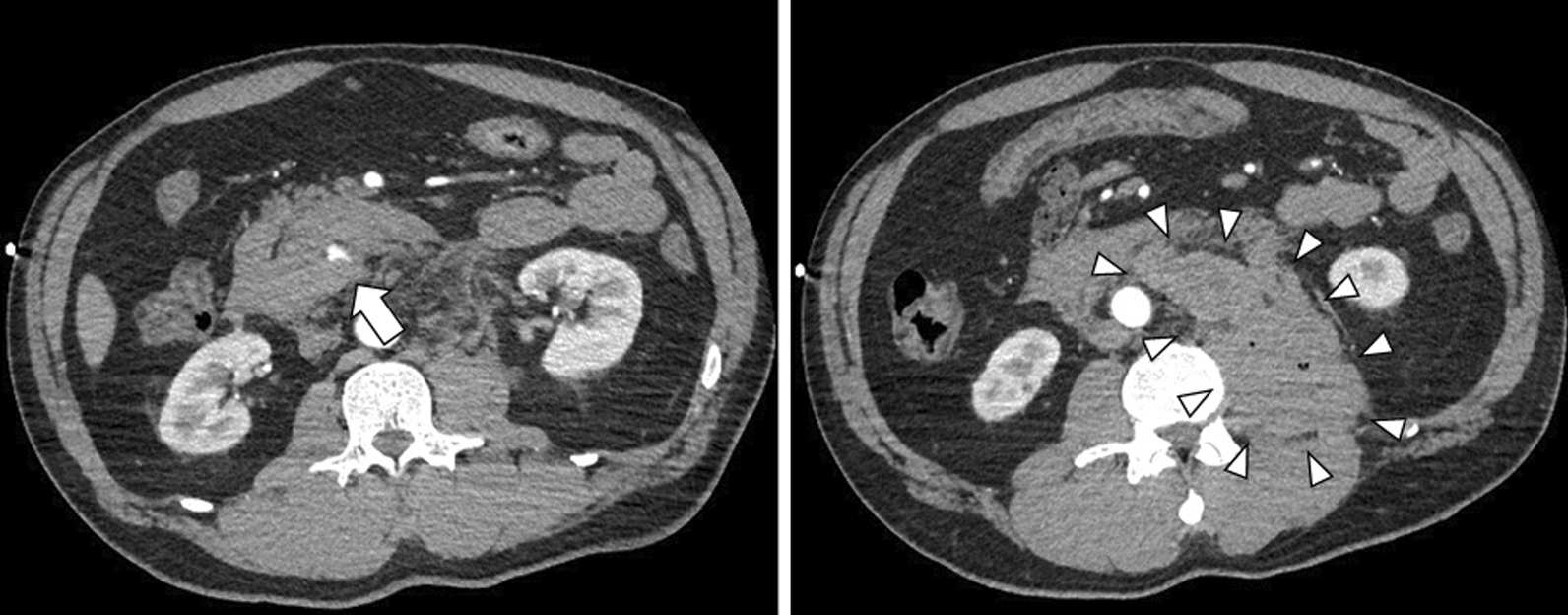
Image showing extravasation of the contrast medium around the pancreatic head (arrow), and hematomas behind the pancreas and in the left psoas muscle (arrowheads).

**Fig. 2:**
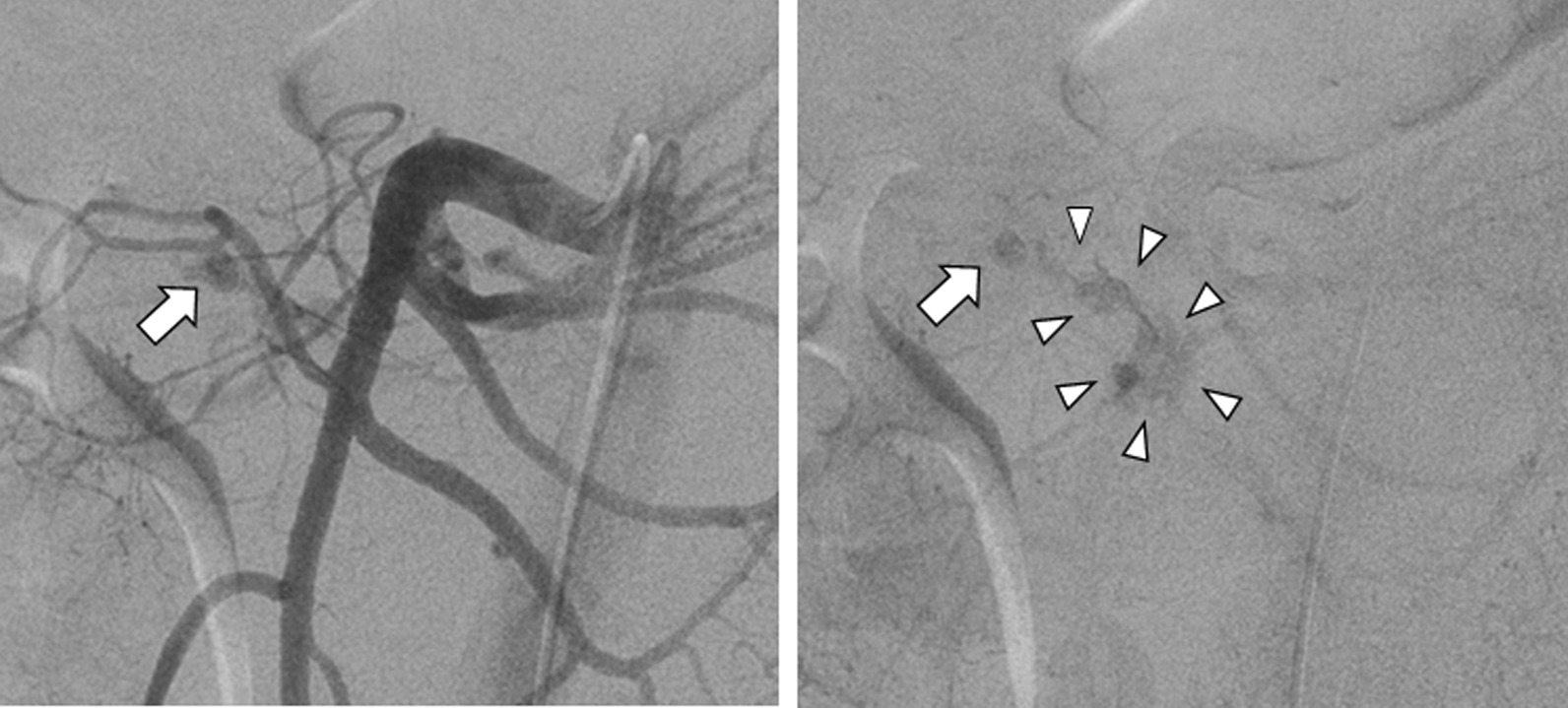
Abdominal arterial angiographic image showing the pseudo aneurysm in the branch of the inferior pancreatoduodenal artery (arrow) and extravasation of the contrast medium from that artery (arrowheads).

A 47-year-old Japanese man, stabbed in the back with a knife, was transferred to our emergency room. He presented with a stab-wound in his left back, and slight tenderness in his abdomen; although his hemodynamic state was unstable, it was improved by a bolus infusion. Upon blood examination, most laboratory parameters were normal, including hemoglobin and coagulation; both serum amylase and lipase levels were within normal ranges (61 U/L and 9 U/L, respectively). Contrast-enhanced computed tomography (CECT) showed extravasation of the contrast medium around the pancreatic head, as well as hematomas behind the pancreas and in the left psoas muscle (Fig. 1); no other visceral or major vascular injuries were presented. We performed abdominal arterial angiography and extravasation of the contrast medium through the inferior pancreatoduodenal artery (IPDA), revealing a pseudo aneurysm in the IPDA branch (Fig. 2). Coil embolization of the IPDA was therefore performed, and the hemodynamic state was stabilized.

On day 1, the serum amylase level was elevated (1366 U/L); however, duct injury was not confirmed via CT. Acute pancreatitis was diagnosed due to pancreatic trauma, and conservative treatment using octreotide was initiated. Additionally, magnetic resonance cholangio-pancreatography was planned to evaluate duct involvement; however, it was acknowledged that the metal clip used in a cholecystectomy performed more than 20 years prior might be contraindicative to magnetic resonance imaging. We therefore selected endoscopic retrograde cholangio-pancreatography (ERCP), as the presence of duct disruption was not completely denied.

**Fig. 3:**
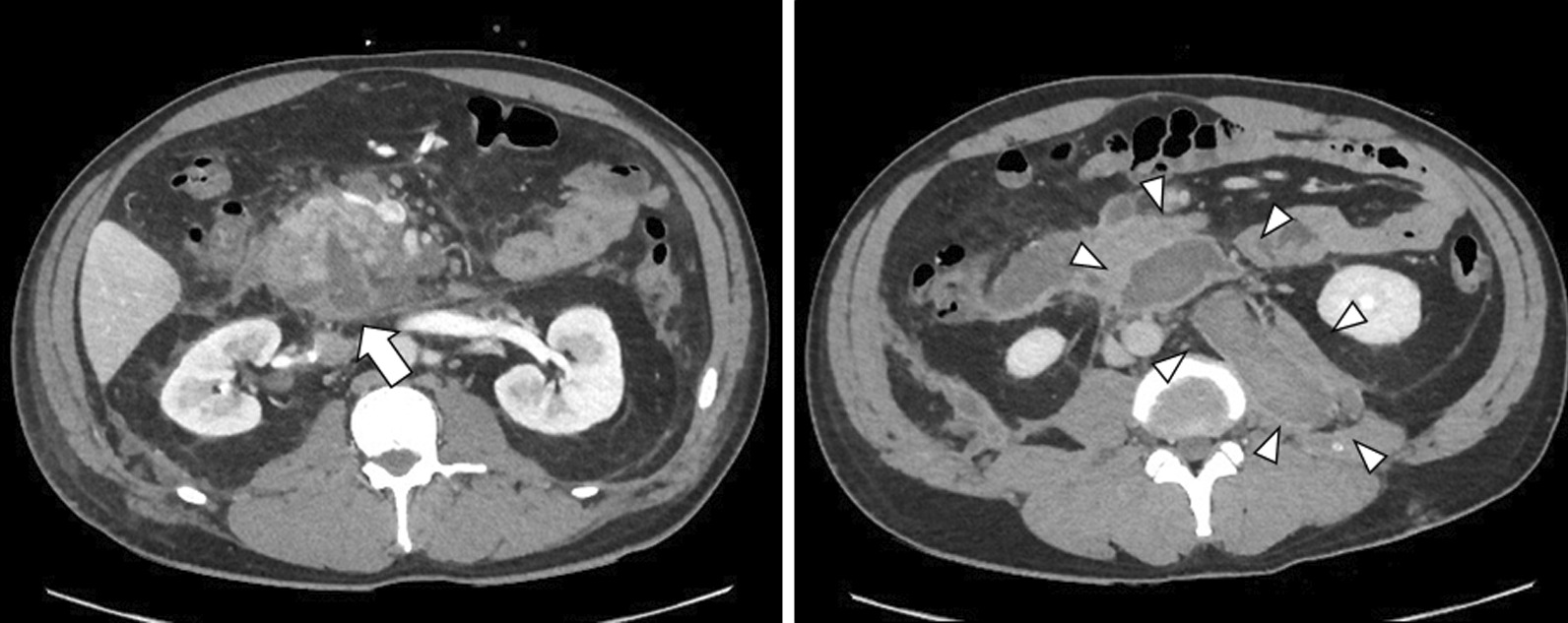
Computed tomographic image showing a wedge-shaped, low-density area in the pancreatic head (arrow), and consecutive pseudocysts behind the pancreas and in the left psoas muscle (arrowheads).

**Fig. 4:**
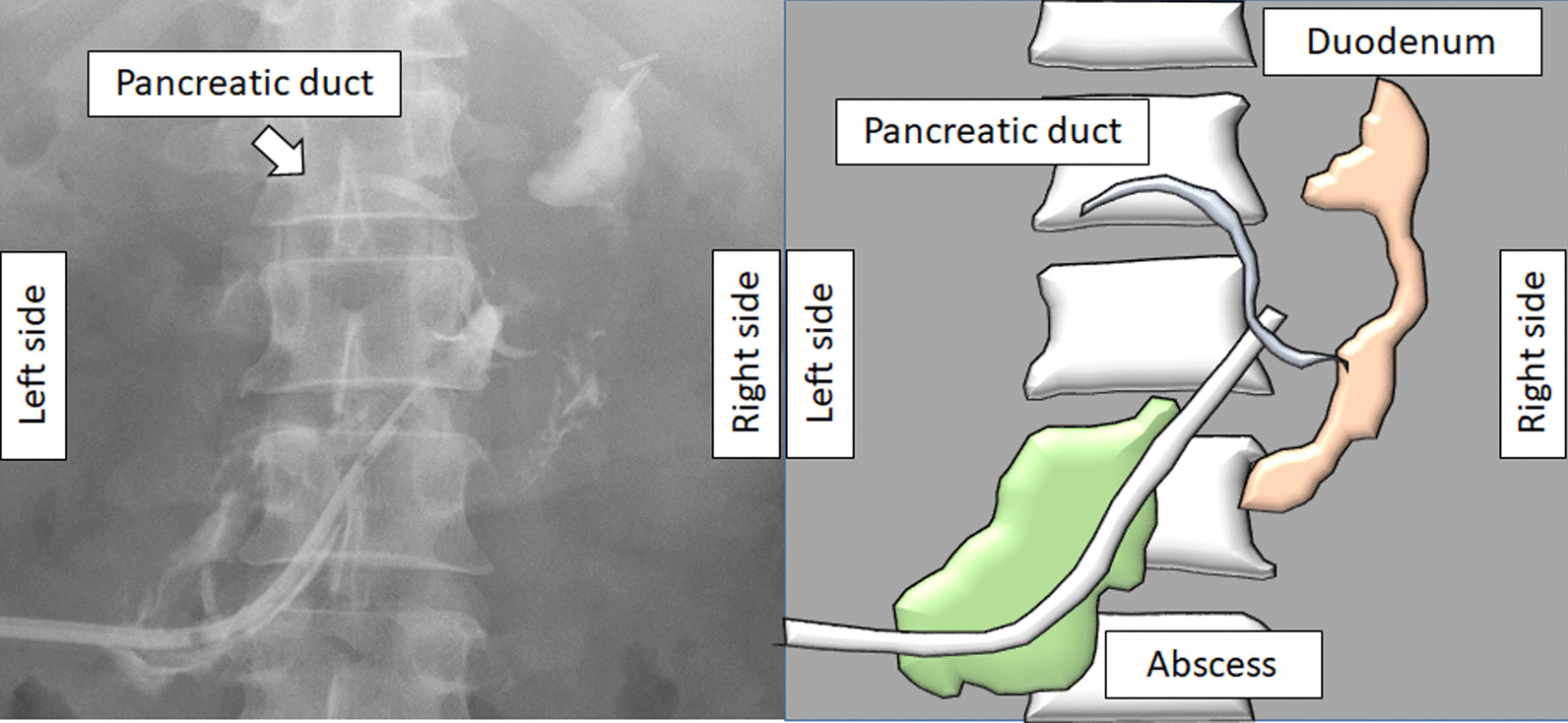
Contrast radiographic image through the percutaneous drain showing the distal part of the main pancreatic duct.

**Fig. 5:**
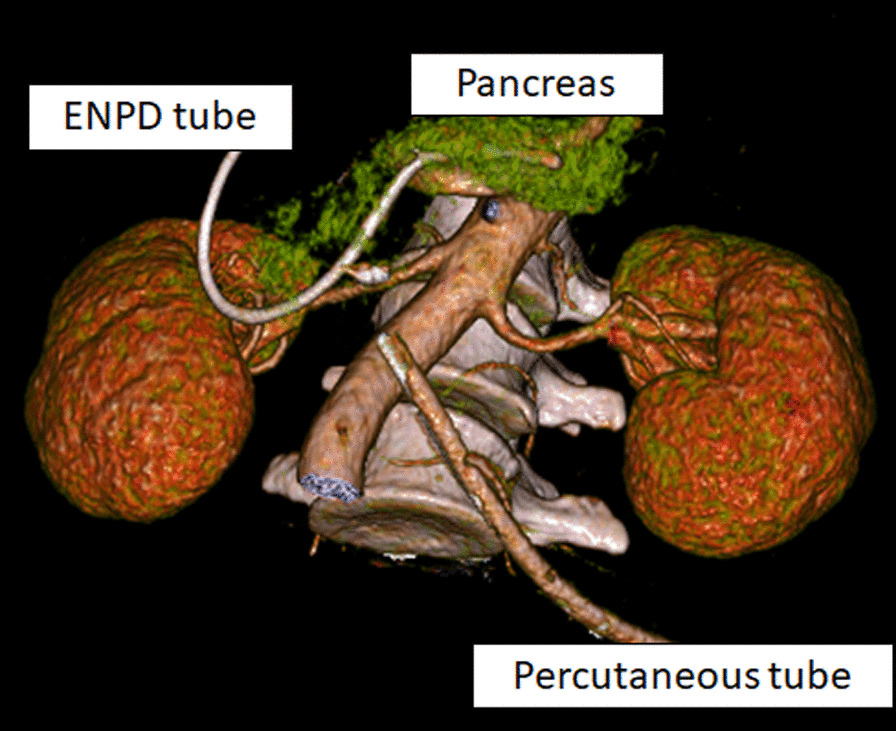
3-Dimensional image of the abdominal computed tomography scan showing the stent across the ductal disruption and percutaneous drainage tube.

**Fig. 6:**
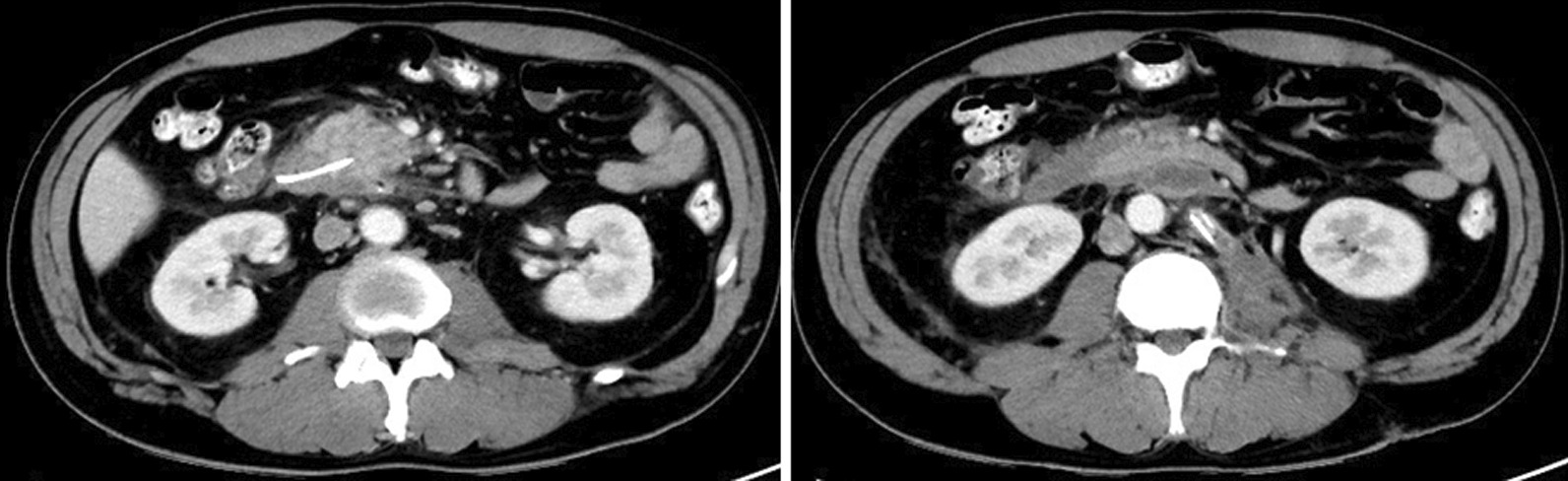
Computed tomographic image showing a marked reduction in the fluid collection after drainage.

On day 5, although ERCP was performed, the scope did not reach Vater’s papilla due to the narrow cavity of the decompressed duodenum. The serum amylase level gradually decreased and then normalized. On day 12, CT revealed a wedge-shaped, low-density area in the pancreatic head, and consecutive pseudocysts behind the pancreas and in the left psoas muscle (Fig. 3). Thereafter, a percutaneous drain was placed through the stab wound, behind the pancreatic head. On day 22, contrast radiography through the percutaneous drain revealed the proximal and distal parts of the main pancreatic duct (Fig. 4); therefore, the patient was diagnosed with AAST grade IV pancreatic injury. On day 26, an endoscopic nasopancreatic drainage (ENPD) tube was inserted across the disruption (Fig. 5), and on day 38, CECT showed a marked reduction in the fluid collection (Fig. 6). The ENPD tube was changed to an endoscopic retrograde pancreatic drainage (ERPD) tube on day 40, and he was discharged on day 61. The ERPD tube was removed 10 months later, and stenosis has not been confirmed on magnetic resonance cholangiopancreatography after 1.5 years.

## Discussion

Pancreatic trauma rarely occurs when compared with other solid organ injuries of the abdomen; the incidence of pancreatic injuries among all types of trauma has been reported as 0.21−0.32% in three databases [6–8]. Among patients with all types of abdominal injuries, 3.1% of pancreatic injury cases were confirmed according to a review of the National Trauma Data Bank [9]. Pancreatic injury is usually associated with other abdominal traumas: liver (15.7%), vascular (15.5%), spleen (9.3%), mesenteric (8.1%), duodenum (5.8%), and kidney (5.4%) [10]. Owing to its location and proximity to other organs and major vascular structures, isolated pancreatic injuries are rare, especially in penetrating trauma; only 3% of penetrating injuries of the pancreas are isolated [10, 11].

Treatment for grade III and IV pancreatic trauma is controversial. The EAST guidelines recommend NOM for grade I and II pancreatic injuries, and operative management for grade III and IV injuries [2]. Indeed, Siboni *et al.* showed that NOM for severe pancreatic trauma such as grade IV or V was associated with higher mortality (nonoperative: 6%; laparotomy alone: 3%; repair/resection: 0%) [9]; however, some researchers have reported that the mortality rate of grade III and IV was not significantly different between the operative and nonoperative treatment groups (13.8% vs. 12.3%, respectively) [10], or between the resection and nonresection groups (15.1% vs. 18.4% in grade III, and 24.0% vs 27.1% in grade IV, respectively) [12]. Additionally, the length of hospital stay was not significantly different between the two groups (32 days vs. 29 days, respectively) [10]. The present case was successfully managed by NOM with endoscopic and percutaneous drainage. Although the EAST guidelines recommend operative treatment for high-grade pancreatic trauma, NOM with the appropriate drainage may be a promising treatment for grade III or IV trauma, especially at facilities with expertise in interventional radiology and endoscopy.

The treatment approaches of NOM include endoscopic ductal stenting alone, percutaneous drainage alone, endoscopic cysto-enterostomy alone, and a combination of the above. Koganti *et al.* reported that NOM with percutaneous drainage alone or endoscopic cyst-enterostomy alone showed a high morbidity rate, with abdominal abscess in 6/10 cases, and pseudocyst formation in 8/10 cases [13]. Kim *et al.* reported that NOM with endoscopic stent insertion alone resulted in pseudocyst formation in 8/11 cases, main pancreatic duct stricture in 4/11 cases, and pancreatic atrophy of the distal part in 3/11 cases [14]. In our case, complications such as abscess formation, pseudocyst formation, and duct stricture were not confirmed after drainage. We believe that the combination of ductal and percutaneous drainage facilitated the success of NOM.

The practical use of octreotide for the management of pancreatic trauma is controversial [15, 16]. The EAST guidelines conditionally recommend routine use of octreotide as a postoperative prophylaxis for traumatic pancreatic injuries to prevent fistula. However, the supporting studies are not well designed and contain a small number of the patients; therefore, further clinical trials are warranted to overcome these limitations.

Regarding pancreatic resection, parenchymal preservation is paramount with respect to endocrine and exocrine functions. After pancreatoduodenectomy (PD), up to 30% of nondiabetic patients develop postoperative, new-onset diabetes, while 14−15.5% experience persistent glucose intolerance for 1−8 years after PD. In addition, exocrine dysfunction was observed during the long-term follow-up post-PD for benign and malignant tumors in 25% and 49% of patients, respectively [17]. If operative treatment is used for grade III/IV pancreatic trauma, it is necessary to preserve pancreatic parenchyma for as long as possible.

Lin * et al.* investigated the long-term outcomes of stent insertion; they described that ductal stricture was a major complication [18]. Abe *et al.* reported the same complication after stent insertion (4); thus, follow-up of post-stenting is warranted for a period. In our case, the ERPD tube was removed after 10 months, and stenosis and atrophy have not been confirmed on magnetic resonance cholangiopancreatography after 1.5 years.

## Conclusions

Here, we reported a case of successfully managed endoscopic pancreatic duct stenting and percutaneous drainage for grade IV pancreatic injury. This suggests that treatment using a combination of endoscopic and percutaneous drainage may avoid the need for operation.

## Data Availability

Not applicable.

## Abbreviations

AAST: The American Association for the Surgery of Trauma
CECT: Contrast-enhanced computed tomography
EAST: East Association of Surgery for Trauma
ENPD: Endoscopic naso-pancreatic drainage
ERCP: Endoscopic retrograde cholangio-pancreatography
ERPD: Endoscopic retrograde pancreatic drainage
IPDA: Inferior pancreatoduodenal artery
NOM: Nonoperative management
PD: Pancreatoduodenectomy
